# Effects of Alu elements on global nucleosome positioning in the human genome

**DOI:** 10.1186/1471-2164-11-309

**Published:** 2010-05-17

**Authors:** Yoshiaki Tanaka, Riu Yamashita, Yutaka Suzuki, Kenta Nakai

**Affiliations:** 1 Department of Medical Genome Sciences, Graduate School of Frontier Sciences, University of Tokyo, 4-6-1 Shirokanedai, Minato-ku, Tokyo 108-8639, Japan; 2 Human Genome Center, Institute of Medical Science, University of Tokyo, 4-6-1 Shirokanedai, Minato-ku, Tokyo 108-8639, Japan; 3 Institute for Bioinformatics Research and Development (BIRD), Japan Science and Technology Agency, 5-3 Yonbancho, Chiyoda-ku, Tokyo 102-0081, Japan; 4 Frontier Research Initiative, Institute of Medical Science, University of Tokyo, 4-6-1 Shirokanedai, Minato-ku, Tokyo 108-8639, Japan

## Abstract

**Background:**

Understanding the genome sequence-specific positioning of nucleosomes is essential to understand various cellular processes, such as transcriptional regulation and replication. As a typical example, the 10-bp periodicity of AA/TT and GC dinucleotides has been reported in several species, but it is still unclear whether this feature can be observed in the whole genomes of all eukaryotes.

**Results:**

With Fourier analysis, we found that this is not the case: 84-bp and 167-bp periodicities are prevalent in primates. The 167-bp periodicity is intriguing because it is almost equal to the sum of the lengths of a nucleosomal unit and its linker region. After masking Alu elements, these periodicities were greatly diminished. Next, using two independent large-scale sets of nucleosome mapping data, we analyzed the distribution of nucleosomes in the vicinity of Alu elements and showed that (1) there are one or two fixed slot(s) for nucleosome positioning within the Alu element and (2) the positioning of neighboring nucleosomes seems to be in phase, more or less, with the presence of Alu elements. Furthermore, (3) these effects of Alu elements on nucleosome positioning are consistent with inactivation of promoter activity in Alu elements.

**Conclusions:**

Our discoveries suggest that the principle governing nucleosome positioning differs greatly across species and that the Alu family is an important factor in primate genomes.

## Background

The genomic DNA of eukaryotes forms chromatin structures with several proteins. Chromatin is composed of nucleosome cores in which 146-147 base pairs (bp) of DNA are wrapped in 1.67 turns around a histone octamer containing two copies each of four core histones: H2A, H2B, H3, and H4 [1]. Another histone (linker histone) binds to about 20 bp of DNA in the linker region flanking the nucleosome core [2,3]. Nucleosomes are involved in various cellular processes, including transcription, because chromatin can limit the accessibility of regulatory sites. For example, it has been reported in several organisms that the nucleosome occupancy rate upstream from transcription start sites (TSSs) is lower than that in other regions [4-12]. Therefore, understanding the mechanism of nucleosome positioning is important for the analysis of transcriptional regulation and promoter functions.

It is known that nucleosome positioning can be affected by DNA sequence. Many previous studies have identified various motifs for nucleosome positioning or inhibition with *in vivo *and *in vitro *experiments [13-18]. It is also known that 10-bp periodic AA/TT or GC dinucleotides are strongly associated with nucleosome positioning in the genomes of several species and in synthetic DNAs [19-22]. Short oligonucleotides occurring at intervals of about 10 bp are associated with the positions of the major grooves or minor grooves facing the histone surface and with the bendability of DNA during nucleosome formation [23]. Using these dependencies, some researchers recently succeeded, more or less, in the computational prediction of nucleosome positions in the genome sequences of several yeasts [24-27]. In particular, Segal et al. explained about 50% of *in vivo *nucleosome positions using a position weight matrix of center-aligned mononucleosome DNA in budding yeast and chicken [28]. The 10-bp periodicity has been observed by Fourier analysis in the genome of nematode, plant, insect and fungus [29].

In recent years, high-throughput sequencing techniques and tiling array experiments have provided an avalanche of nucleosomal DNA location information in the human [8-10], fly [11,30], nematode [7,20], and budding yeast genomes [4-6,12]. Schones et al. demonstrated nucleosomal reorganization during the activation of human T cells using a large number of nucleosomal DNAs, which were massively sequenced with a new-generation sequencer. Lee et al. and Shivaswamy et al. showed that about 70%-80% of the whole genome of budding yeast is occupied by nucleosomes. These large-scale experiments make it possible to analyze the sequence dependencies of global nucleosomal positioning across a wide range of organisms.

In this study, we first asked whether the reported periodic motifs can widely affect *in vivo *nucleosome locations through the whole genomes of all eukaryotes. Using Fourier analysis, the spectrum of primate genomes does not exhibit clear peaks with a 10-bp periodicity: strong and wide 84-bp and 167-bp periodicities are observed, instead. These periodicities, which may be associated with the length of DNA wrapping core histones and the linker histone, mainly originate from Alu repetitive elements, as their strength decreased markedly in Alu-masked genomes.

The Alu family are primate-specific short interspersed elements (SINEs), and constitute the most prevalent repetitive element in the human genome [31]. Alu elements are categorized into two groups: monomers and dimers. A typical dimeric Alu is about 300 bp long, and is composed of two distinct GC- and CpG-rich monomers flanking an A-rich region and a poly(A) tract. Monomeric Alu elements consist of two classes: free left Alu monomers (FLAM) and free right Alu monomers (FRAM), corresponding to each monomer in a dimer. The left monomer is slightly shorter than the right one [32]. Although a few Alu elements in promoters are reported to affect their downstream gene expression [33,34], most of them are silent in cells. Thus, specific positioning of nucleosomes on Alu elements may be important in masking unnecessary effects of Alu's to nearby genes. DNase I nicking analyses have demonstrated that several dimeric Alu elements have some affinity to core histones [35,36]. According to these studies, two nucleosomes are formed in both sides of the central A-tract of Alu elements.

Using large-scale nucleosome mapping data, we observed that such specific positioning occurs globally in the entire genome. We further showed that nucleosomes are also arranged in nucleosomal repeat lengths (about 170-200 bp) around them. Our discoveries should be useful in the prediction of nucleosomal positions in primates and the analysis of transcriptional regulation.

## Results

### Genome-wide nucleotide periodicity

We analyzed the genome-wide nucleotide periodicity in 16 different species (See additional file 1) under the assumption that if the 10-bp periodic nucleotides are the main factor determining nucleosome positioning, peaks of this periodicity should also be observed in the whole genome. The strength of the periodicity was calculated for 12 kinds of mono- and di-nucleotide steps using Fourier analysis [21,37].

**Figure 1:**
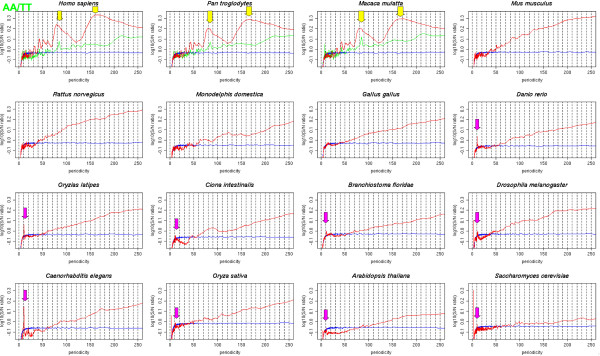
**Strength of the genome-wide periodicity from 2 bp to 250 bp for the AA/TT dinucleotide step**. Red, blue, and green lines are drawn from the results for the whole genome, randomly generated DNA, and Alu-masked genome sequences, respectively. Pink arrows represent peaks of 10-bp periodicity, and yellow arrows represent peaks of 84-bp or 167-bp periodicity.

In consistent with a previous study [29], clear spikes with a periodicity of about 10 bp or 11 bp were observed in fishes, chordates, an insect, a nematode, plants, and a fungus with the AA/TT dinucleotide step (Figure 1). In the nematode genome, another small peak with a periodicity of about 9 bp was observed (See additional file 2). The spectra of these organisms calculated with other nucleotide steps also showed clear peaks of about 9-bp, 10-bp, or 11-bp periodicity. In the chicken genome, weak peaks of 10-bp periodicity were observed only for the A/T and G/C steps. However, no clear peaks with these periodicities were observed in mammals. In contrast, two large and wide peaks centered at about 84 bp and 167 bp were observed in the human, chimpanzee, and rhesus genomes. These primate-specific peaks were clearly observed in the AA/TT, A/T and G/C steps, and were especially remarkable in the AA/TT step (See additional file 3). The value of 167 bp corresponds approximately to the sum of the length of a nucleosome core (147 bp) and the length of the linker-histone-binding region (20 bp), and 84 bp corresponds to about half this length. This observation suggests that these periodicities are related to nucleosome positioning in primate genomes.

Because it is known that some repetitive elements are detectable by nucleotide periodicities [38], we next analyzed the genome-wide periodicity while masking all Alu elements, which are primate-specific SINEs detected by RepeatMasker. The strengths of the 84-bp and 167-bp periodicities in the Alu-masked primate genomes were remarkably lower than those in the nonmasked genomes (green lines in Figure 1 and additional file 3). Although weak and sharp peaks of 84-bp or 167-bp periodicity still remained in some nucleotide steps, they were possibly derived from Alu elements that had not been identified by RepeatMasker or from some other genome features. Thus, it is clear that these 84-bp and 167-bp periodicities mainly originate from Alu repetitive elements.

### Effects of Alu elements on nucleosome positioning

**Figure 2:**
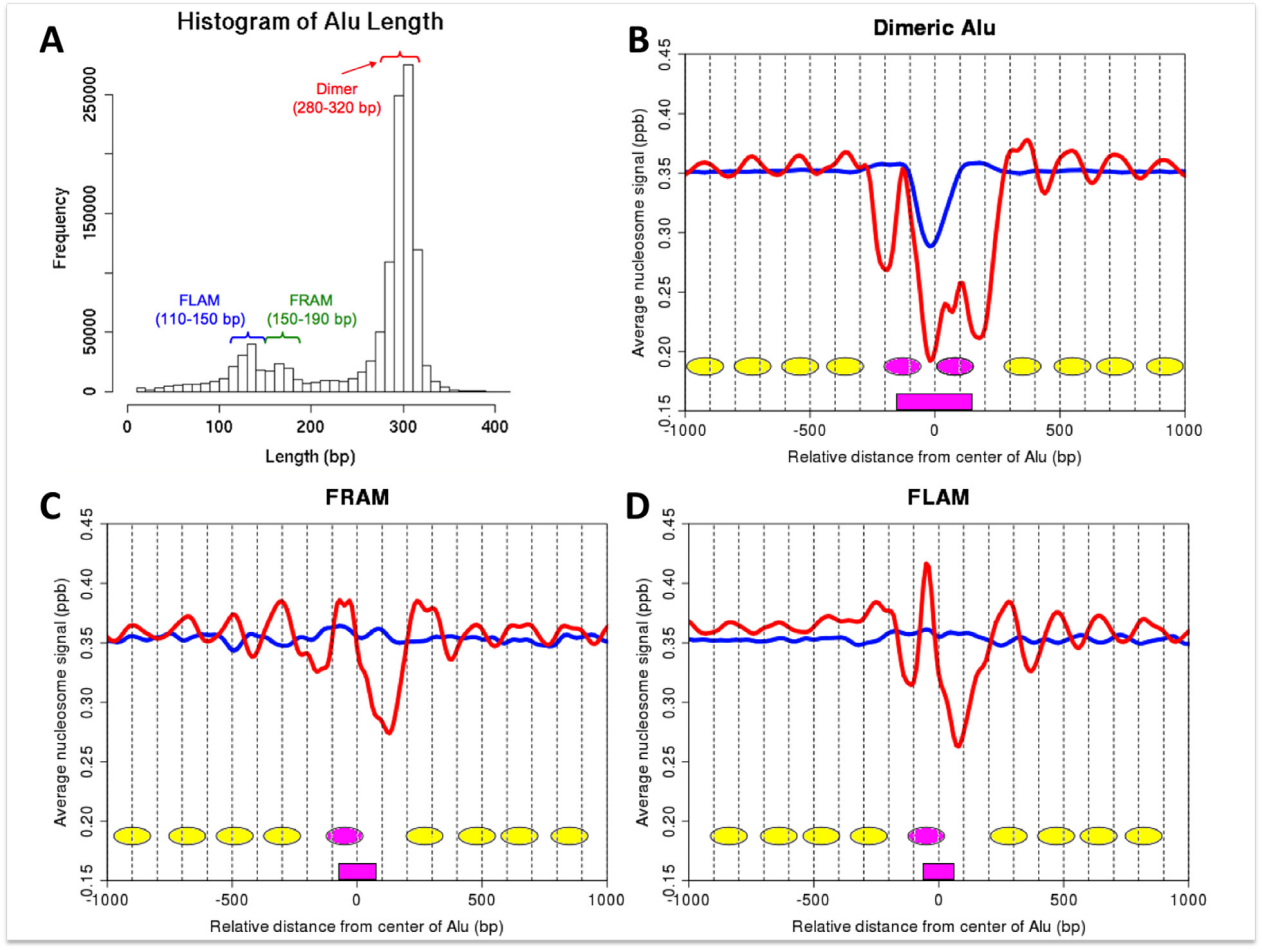
**Analysis of nucleosome distributions within and around Alu elements**. (A) Histogram of the lengths of all Alu elements detected by RepeatMasker. Nucleosome distributions with respect to (B) dimeric Alu elements, (C) FLAMs, and (D) FRAMs are shown. Red and blue lines indicate the distributions in nucleosome DNA tags and random fragments, respectively. Nucleosome positions estimated from the distributions are depicted as pink (within Alu elements) or yellow ovals (around Alu elements).

To confirm the relationship between Alu elements and nucleosome positioning in the human genome, we analyzed the distribution of nucleosomes within and around the Alu elements. In this study, the Alu family was categorized into three classes: dimer, FLAM, and FRAM. After removing incomplete Alu elements, we identified 763,485 dimers of 280-320 bp in length, 47,815 FLAMs of 110-150 bp, and 17,255 FRAMs of 150-190 bp (Figure 2A). These occupy about 7.42%, 0.20% and 0.09% of the whole human genome, respectively.

We used the data of paired-end nucleosomal DNA tags (sequenced fragments) from HEK293 cells made by the micrococcal nuclease (MNase) digestion and by the sequencing with the Illumina GA-II sequencers (Irie et al., submitted). In total, 27,756,155 paired-end nucleosome DNA tags were mapped on the human genome (Table 1). More than 90% (26,340,652 tags) of them showed unique positions, and about 5.91% (1,639,194 tags) were assigned within the Alu elements. By aligning the nucleosome signal (i.e., the smoothed positional distribution of mapped tag frequency; see Methods) to the central position of each type of Alu elements, we found one clear peak on the left side and another weaker peak on the right side in dimeric Alu elements (Figure 2B) as well as a single clear peak in both FLAMs and FRAMs (Figure 2C and 2D, respectively). On the other hand, large troughs of the nucleosome signal were observed near the A-rich regions (See additional file 4). In the dimeric Alu elements, the signal intensity of the left peak is not so different from that of randomly extracted fragments (used as control) and the intensity of the right peak is much smaller than the normal level. These results indicate that the positions where the nucleosome structure is formed within dimeric Alu elements are very limited; of the two possible slots, one is used at the normal level while the other is disfavored. On the other hand, monomeric Alu elements have a single possible slot.

**Table 1:** Mapping results of nucleosome DNA tags

	Unique		Multi			Total
	Alu	Others	Complete	Partial	None	
Nucleosomal DNA tags	1,639,194	24,701,458	95,382	86,169	1,233,952	27,756,155
	5.91%	88.99%	0.34%	0.31%	4.45%	100.00%
Random fragments	2,220,784	26,040,905	164,102	148,157	1,426,052	30,000,000
	7.40%	86.80%	0.55%	0.49%	4.75%	100.00%

**Figure 3:**
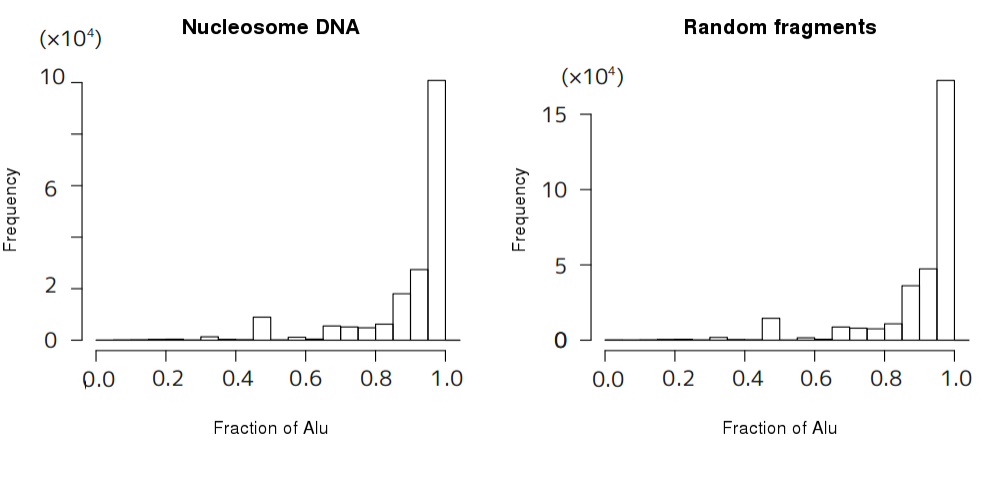
**Histograms of the fraction of Alu elements overlapping with the multi-hit positions of (A) nucleosome tags and (B) random fragments**.

The 181,551 multi-hit tags that overlap with Alu elements were not included in the above result (Table 1). For about a half of them (95,382 tags), all of their multiple hits were mapped within Alu elements while for 80% of them (146,477 tags) more than 85% of their multiple hits overlapped with Alu elements (Figure 3). Then, from the above result only, there may remain some concern that the observed slots are only artifacts caused by the removal of tags mapped to multiple locations. Thus, we randomly chose one from each of multi-hit tags that were mapped within the Alu region and redrew the distribution of nucleosome signal around Alu elements (Additional file 5). Similarly with Figures 2B-D, the nucleosome distribution shows two or one slots in Alu elements and lower occupancy of nucleosome is observed in the right arm of the dimeric Alu's. When we repeated the same procedure using the muti-hit tags more than 85% of which overlap with Alu elements, similar distributions were observed (Additional file 5). Furthermore, standard deviation of the relative distances of multiple hits from the center of nearest Alu elements for each tag was only 2.248 bp on average. Overall, we concluded that the nucleosome slots in Alu elements are not artifacts.

We also detected phased nucleosome positioning at approximately the nucleosome repeat length (about 170-200 bp) around the Alu elements (yellow ovals in Figure 2) [39]. This phased frequency extended to both orientations and became weaker as the site is more distant from Alu elements. These observations suggest that Alu elements contain nucleosomes in specific positions, and also influence the positioning of neighboring nucleosomes.

### Validation of the positioning of neighboring nucleosomes by independent data

**Figure 4:**
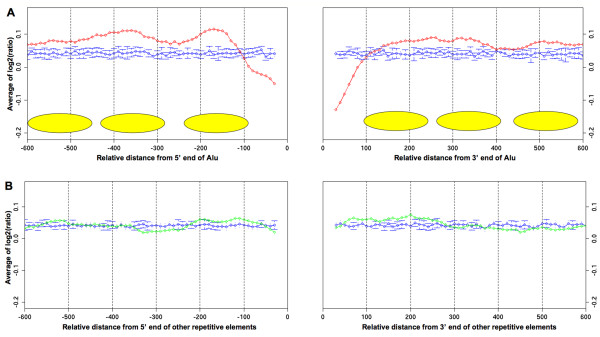
**Distributions of hybridization signals around (A) Alu elements and (B) other repetitive elements**. Red and green lines show the average hybridization values of probes flanking Alu elements and other repetitive elements, respectively. The blue line shows the background means and standard deviations calculated from 10 randomized data sets. Estimated nucleosome positions are represented by yellow ovals.

To verify this suggestion with another independent experiment, we analyzed the distribution of nucleosome locations using the data from high-resolution tiling arrays for 3,962 human promoters in seven different cell types [9]. Each probe in every array was aligned by both edges of the Alu elements, including both monomers and dimers, and the signals were averaged at each position for all arrays. The hybridization signals were significantly higher than the background signal at regions just upstream and downstream from Alu elements (Figure 4A). This result indicates that nucleosomes are preferentially located in the neighborhood of Alu elements. This conclusion remains unchanged in the data for each cell type, except A375 (See additional file 6). Furthermore, in a region of about 100 bp immediately upstream and downstream from the Alu elements, the signals were lower than the background signal. This tendency was also observed in the distribution of nucleosome centers (Figure 2B-D). These results imply that the regions immediately upstream and downstream from Alu elements are likely to be preferentially used as linker regions. Although we analyzed the signals flanking other repetitive elements, there was little difference from the background signal (Figure 4B and additional file 6), indicating that the effect on the positioning of neighboring nucleosomes is specific to Alu elements.

Considering these data together, it is clear that the Alu family significantly affects nucleosome positioning in the human genome.

### Relationship between nucleosome positioning and gene expression

**Figure 5:**
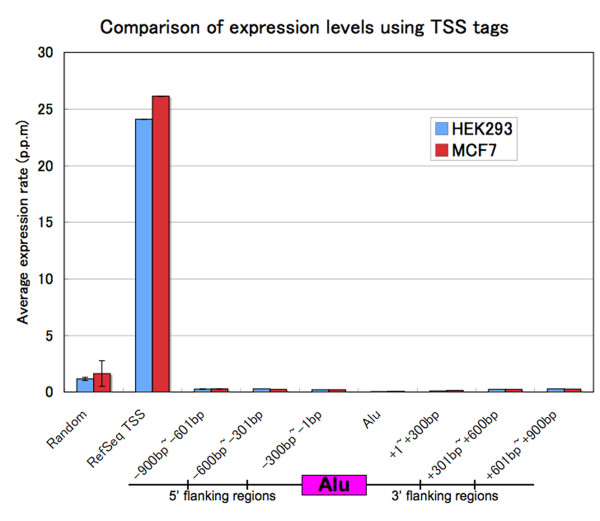
**Comparison of expression levels using precise 5'-end cDNA tags**. Red and blue bars are drawn from the results of HEK293 and MCF7, respectively. In random site category, averages and standard deviations (error bars) from ten sites are shown.

It has been suggested that nucleosomes are predominant in promoters of unexpressed genes [8,9]. Therefore, the observed restriction of nucleosome positioning within and around Alu elements may influence their promoter functions *in vivo*. To verify this possibility, we calculated the average expression rate from the number of precise 5'-end cDNA tags mapped within Alu elements. As controls, the rates for 5'-end tags mapped in regions around RefSeq TSSs and randomly selected regions were also calculated. Precise 5'-end tags for HEK293 and MCF7 cells were previously detected by the oligo-capping method and were sequenced with an Illumina Solexa sequencer [40]. The average expression rates in an Alu element were about 0.045 and 0.062 parts per million (p.p.m) in each cell type, respectively (Figure 5). In contrast, the expression rates in RefSeq TSSs were more than 20 (24.09 and 26.13 p.p.m, respectively), remarkably higher than those in the Alu elements, and those at random sites were also higher than those in Alu elements (1.16 and 1.62 p.p.m, respectively). We further calculated the rates in the flanking regions: [-900, -601], [-600, -301], [-300-1], [+1, +300], [+301, +600] and [+601, +900]; -1 and +1 indicate 5' and 3' end of Alu elements, respectively. The expression rates at the flanking regions are slightly higher than those in Alu elements (from 0.099 to 0.276 p.p.m in HEK293 and from 0.128 to 0.270 p.p.m in MCF7), but are significantly lower than those at random sites. These results suggest that most Alu elements do not have the activity of promoter.

## Discussion

Alu elements are distributed only in primates, and comprise about 10% of the whole human genome [41]. In this study, we showed that nucleosome positioning is significantly influenced within and around Alu elements using large-scale nucleosome mapping data. Englander et al. previously reported that dimeric Alu elements have a capacity containing two nucleosomes [35,36]. These positions are consistent with the two peaks observed in Figure 2B. Since DNase I nicking pattern in their experiment is less clear in the right arm compared with the left arm of dimeric Alu elements, the low occupancy in the right arm observed in our analysis may be caused by the lower stability of nucleosome positioning. From a massive number of nucleosomal DNA sequence data, we demonstrated that neighboring nucleosomes tend to be arranged with regular intervals. Nucleosome signals from the tiling array data showed some preference for nucleosome positioning just around Alu elements. The A-rich sequences observed in low nucleosome density regions are known as a motif for inhibiting nucleosome formation [21]. From our analysis based on two independent data sets, we conclude that the lower nucleosome signals at the boundary of Alu elements affect the arrangement of nucleosome positioning around them. Although nucleosome depletions are often observed in the promoters of active genes, few TSS tags could be mapped in Alu elements and their flanking regions. It is possible that restriction of Alu elements is regulated by other epigenetic factors. This hypothesis is supported by two previous reports that 76.2% of CpG sites in Alu elements are completely methylated [42], and that methylation of H3 lysine 9 is enriched in Alu elements [43].

The genome-wide periodicity of 16 organisms showed differences in their sequence dependence. Peaks with 10-bp periodicity were observed in organisms from budding yeast to chicken. Our results are in agreement with those of Segal et al., who showed that the nucleosome locations in budding yeast can be predicted with the model constructed from chicken nucleosomal DNA. In some organisms, periodicities of about 9 bp and 11 bp have been found. These periodicities might be related to the minimum and maximum periods of the double helix of DNA in nucleosomes because its observed value varies from 9.4 bp to 10.9 bp [44]. Furthermore, Gupta et al. previously showed 3-bp periodicities of CG and GC dinucleotides are overrepresented in nucleosome-forming sequences in promoters and ENCODE regions [45]. In our results, peaks of CG 3-bp periodicities are found in many species, but not in primates, suggesting that these sequence dependencies may not effect across the whole primate genomes.

It is surprising that the sequence features governing global nucleosomal positioning are quite different among organisms, even though the core histone protein families are highly conserved among species [46]. Although it has been reported that a set of DNA molecules with the highest affinity with the histone octamer in an *in vitro *selection assay showed a 10-bp periodicity of mono- or dinucleotide steps [37], our result suggests that they may not use these features as the major mechanism of nucleosomal positioning, to allow flexibility of chromatin remodeling and complex transcription regulation. We showed that a significant part of nucleosome positioning in the primate genomes can be explained by 84-bp and 167-bp periodicities, which were previously reported in human chromosomes 21 and 22 by a similar method [29]. Here, we confirmed that these periodicities are specific to primates because they are due to primate-specific Alu elements.

## Conclusions

Overall, our study provides an important clue to understanding the whole chromatin composition of the human genome. We hope that our discoveries will extend our understanding of the nucleosomal organization in primate genomes, and contribute essential knowledge about the complexities of transcriptional regulation.

## Methods

### Collection of genome sequences and nucleosome mapping data

Genome sequences were downloaded from UCSC, The institute for Genomic Research (TIGR), RIKEN, and *Saccharomyces *genome database (SGD) (See additional file 1). All positions of repetitive elements and RefSeq genes were obtained from "rmsk" files and the "refGene.txt" file in the UCSC database, respectively.

The tiling array data for human promoters (GSE6385) were downloaded from Gene Expression Omnibus http://www.ncbi.nlm.nih.gov/geo/[9]. About 40 million raw sequence reads of precise TSSs from HEK293 and MCF7 cells were obtained from DBTSS http://dbtss.hgc.jp/[40].

### Fourier analysis of DNA sequences

For each species, 10,000 fragments of length 8,193 (2^13 ^+ 1) bp were randomly extracted from the whole-genome sequence without gap regions ("N" characters). As a control, we generated 10,000 random DNA sequences that had the same base composition as the genome sequence of each organism. To apply discrete Fourier transformations to DNA sequences, we transformed the nucleotide steps to binary codes. For two mononucleotide steps and 10 dinucleotide steps, the target step γ; was converted to 1, and the other steps were converted to 0. For example, if γ is AA/TT, a DNA sequence "CTTGAAT" is changed to the binary sequence "010010".

The power spectrum *F*_*γ *_*(n) *of the binary sequence *B*_*γ *_*(k) *(*k *= *0, 1, ..., N - 1*), whose length is *N*, is defined as follows:

where *j*^2 ^= -1, *n *= 0, 1, 2, ..., (*N*/2) - 1, and *W(k) *is a window function. A window function was used for noise reduction at high frequency. In this study, we used the Welch window [47], defined as follows:

To compare the spectra of various genomes, we used the signal-to-noise (S/N) ratio, which is the ratio of each periodicity signal to the background noise of a DNA sequence. The ratio was calculated by the power spectrum *F*_*γ *_*(n) *as follows:

The background noise is defined as the average power spectrum. The S/N ratio *R*_*γ *_*(n) *is interpreted as the relative strength of the periodicity *p *(= *N*/*n*) [38,48,49]. The S/N ratios were calculated for all DNA fragments, and were averaged for each organism. The Fourier transform was calculated with the FFTW3 library [50].

### Analysis of nucleosome distributions within and around Alu elements

In this study, the Alu family was categorized into three classes: dimer Alu, FLAM, and FRAM, based on the "repName" field (See additional file 7). We also restricted the length of each Alu class to 280-320 bp, 110-150 bp, and 150-190 bp, respectively. Sequences that were longer or shorter than these lengths were discarded.

The paired-end nucleosomal DNA tags were taken from HEK293 cells by the micrococcal nuclease (MNase) digestion and by the sequencing with the Illumina GA-II sequencers (Irie et al., submitted). All 36-bp nucleosome reads were mapped to the human genome (chr1-22, chrX and chrY) using Bowtie (version 0.12.2) with the option: "-f -I 122 -X 172 -n 0 -a --best --strata --chunkmbs 2048" [51]. Because the 5' ends of nucleosomal DNA were sequenced on both strands, their midpoint was presumed to be the positions of the nucleosome core centers. Since the digestion of MNase does not always produce complete 147-bp DNA fragments, we further calculated the nucleosome signal *S(i)*, which is introduced as the "coarse-grain smoothing" in [52]. As background, 30 million 36-bp paired-end genome fragments, whose interval is 147 bp, were randomly extracted and were also mapped to the human genome by Bowtie. In the same way, we deduced the background signal *S*_*back*_*(i) *from the uniquely mapped random fragments. The signals, *S(i) *and *S*_*back*_*(i)*, were normalized with the p.p.b. (parts per billion) unit.

### Analysis of nucleosome signals from tiling array data

In the human promoter tiling array [9], we used probes whose center positions were within 600 bp of Alu elements. We also selected other repetitive elements (all repetitive elements except Alu in the UCSC annotation) that were the same size as the Alu elements, and probes within 600 bp of them were extracted similarly. Each probe was sorted by the distance from the 5' end or the 3' end of these repetitive elements. The hybridization values of the probes at each distance were averaged in each cell type or in all cells. As the background of the signals, we shuffled the hybridization values of all probes in the tiling array. This shuffling was repeated 10 times. Averages and standard deviations were calculated from the 10 randomized data sets and were represented by points and error bars, respectively.

### Average expression level of genes

All 24-25-bp precise 5'-end cDNA tags were mapped in Alu family regions, corresponding to ± 150 bp from TSSs of 25,892 RefSeq genes and ± 150 bp from 25,892 randomly selected sites 24 bp downstream, using SeqMap (See additional file 8) [53]. The average expression rate was calculated as p.p.m for each category:

To obtain the random sites, we repeated the calculation of this rate 10 times, and calculated the means and standard deviations of the results.

## Authors' contributions

YT and KN conceived the study and wrote the paper. YT and RY designed the bioinformatics analyses. YS provided the unpublished sequence data of paired-end nucleosomal DNA. YT obtained and analyzed data. All authors read and approved the final manuscript.

## Supplementary Material

Additional file 1 **List of genome sequences used in this study**. Sources and versions of genome sequences are listed. Click here for file

Additional file 2 **Degree of the genome-wide nucleotide periodicity of mono- and di-nucleotide steps from 2 bp to 22 bp**. For each mono-/di-nucleotide step, the degree of the nucleotide periodicity within the ranges of 2-22 bp is shown. Click here for file

Additional file 3 **Degree of the genome-wide nucleotide periodicity of mono- and di-nucleotide steps from 2 bp to 250 bp**. For each mono-/di-nucleotide step, the degree of the nucleotide periodicity within the ranges of 2-250 bp is shown. Click here for file

Additional file 4 **Frequency of mononucleotides within and around Alu elements**. Distribution of mononucleotides within and around Alu elements is shown. Red, green, blue and cyan lines represent the frequency of adenine, cytosine, guanine and thymine, respectively. Click here for file

Additional file 5 **Nucleosome distribution around Alu elements with multi-hit tags considered**. Nucleosome distribution around dimeric Alu elements, FLAMs, and FRAMs are shown. In this data, multi-hit tags are included. Click here for file

Additional file 6 **Distributions of hybridization signals around (A) Alu elements and (B) other repetitive elements**. In each cell type (A375, IMR90, MALME, MCF7, MEC, PM or T47D) or in all cells, average hybridization signals around repetitive elements are shown. Click here for file

Additional file 7 **List of "repName" used for the classification of Alu family**. Correspondence between the names of repeats ("repName" in UCSC) and the three Alu types (dimeric Alu elements, FLAMs and FRAMs) are listed. Click here for file

Additional file 8 **Strategy of detection of sequence tags within ± 150 bp of TSSs**. Green arrows represent DNA sequences used as reference sequences. The orientation of the arrow shows the strand. Click here for file
