# Supplementary material for: Effects of Alu elements on global nucleosome positioning in the human genome
Source: BMC Genomics. 2010 May 17;11:309. doi: 10.1186/1471-2164-11-309 (PMC2878307; doi:10.1186/1471-2164-11-309)
Supplement: Additional file 3 — Degree of the genome-wide nucleotide periodicity of mono- and di-nucleotide steps from 2 bp to 250 bp. For each mono-/di-nucleotide step, the degree of the nucleotide periodicity within the ranges of 2-250 bp is shown. [file 1471-2164-11-309-S3.PDF]

A. AG/CT step

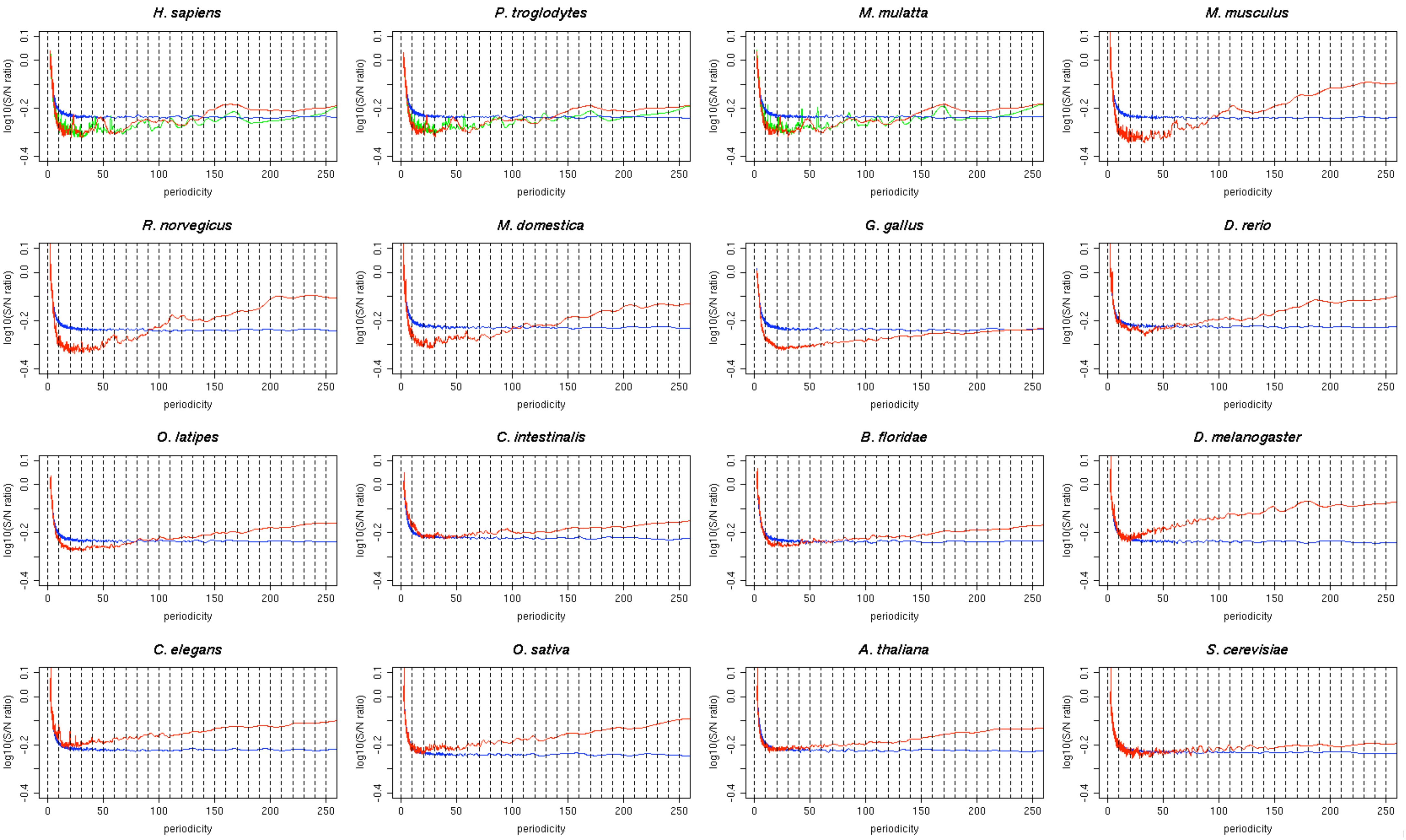
H. sapiens
P. troglodytes
M. mulatta
M. musculus
R. norvegicus
M. domestica
G. gallus
D. rerio
O. latipes
C. intestinalis
B. floridae
D. melanogaster
C. elegans
O. sativa
A. thaliana
S. cerevisiae
log10(S/N ratio)
periodicity

B. AT step

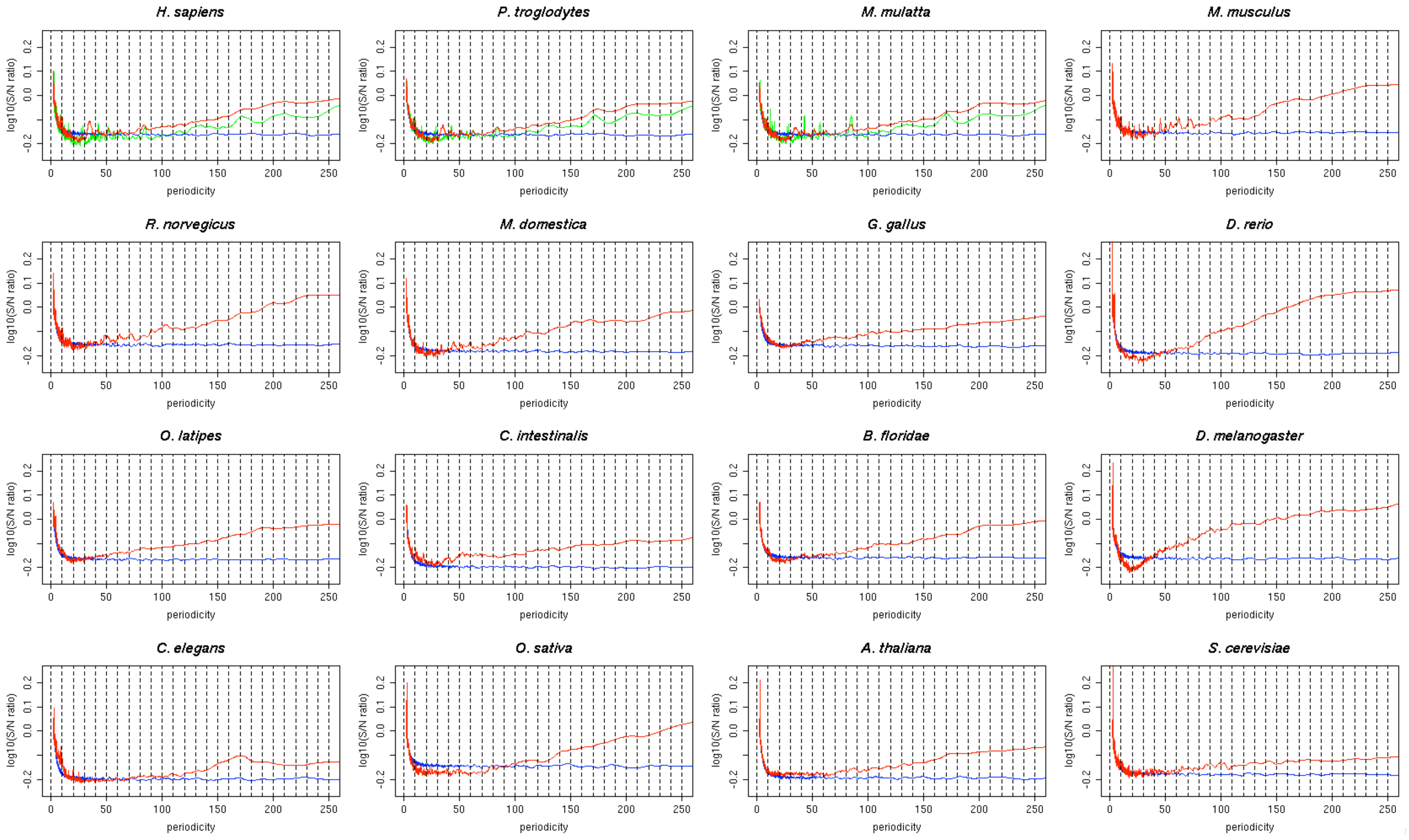
H. sapiens
P. troglodytes
M. mulatta
M. musculus
R. norvegicus
M. domestica
G. gallus
D. rerio
O. latipes
C. intestinalis
B. floridae
D. melanogaster
C. elegans
O. sativa
A. thaliana
S. cerevisiae
log10(S/N ratio)
periodicity
0.2
0.1
0.0
-0.2
0
50
100
150
200
250

C. CG step

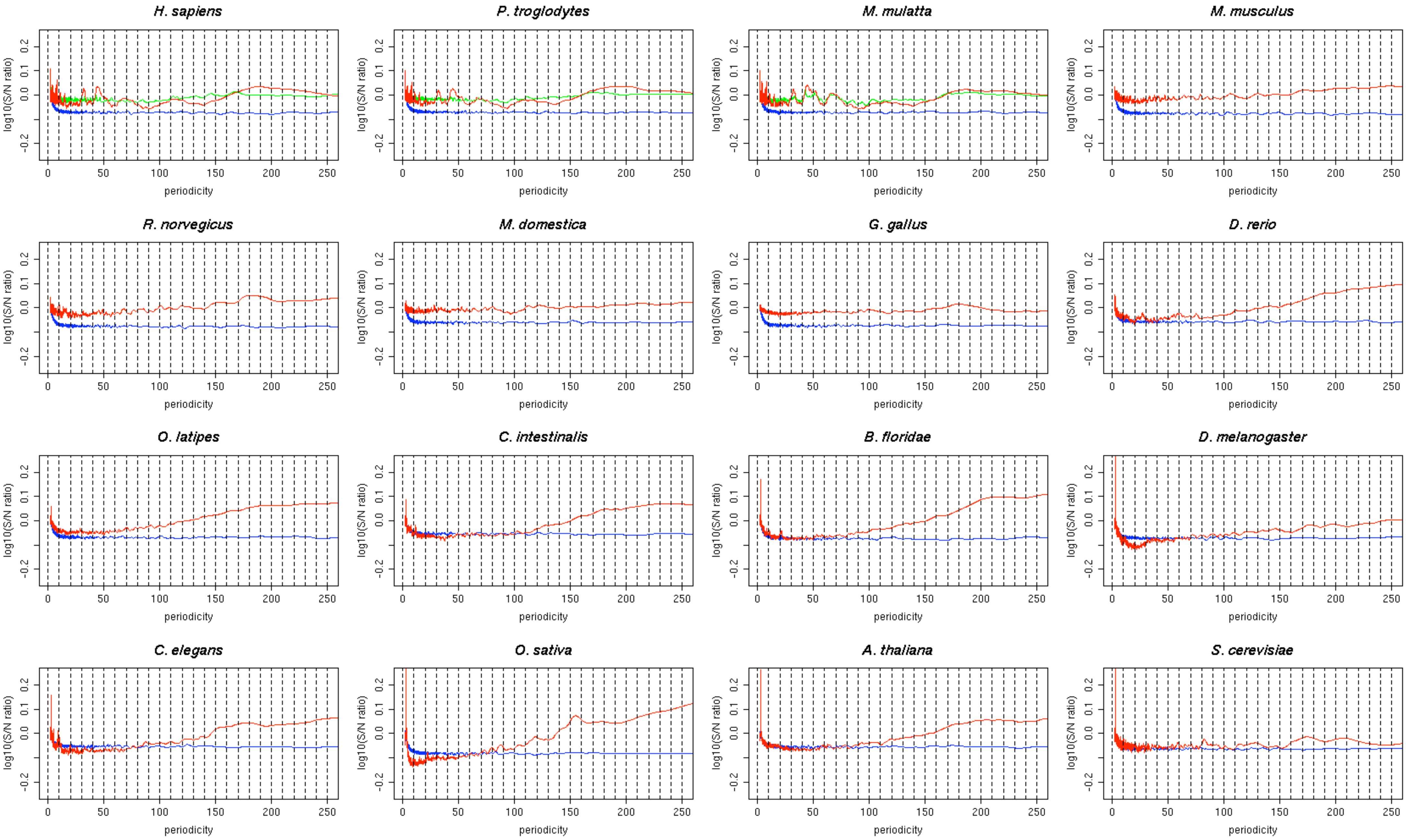
H. sapiens
P. troglodytes
M. mulatta
M. musculus
R. norvegicus
M. domestica
G. gallus
D. rerio
O. latipes
C. intestinalis
B. floridae
D. melanogaster
C. elegans
O. sativa
A. thaliana
S. cerevisiae
log10(S/N ratio)
periodicity
-0.2
0.0
0.1
0.2
0
50
100
150
200
250

D. GA/TC step

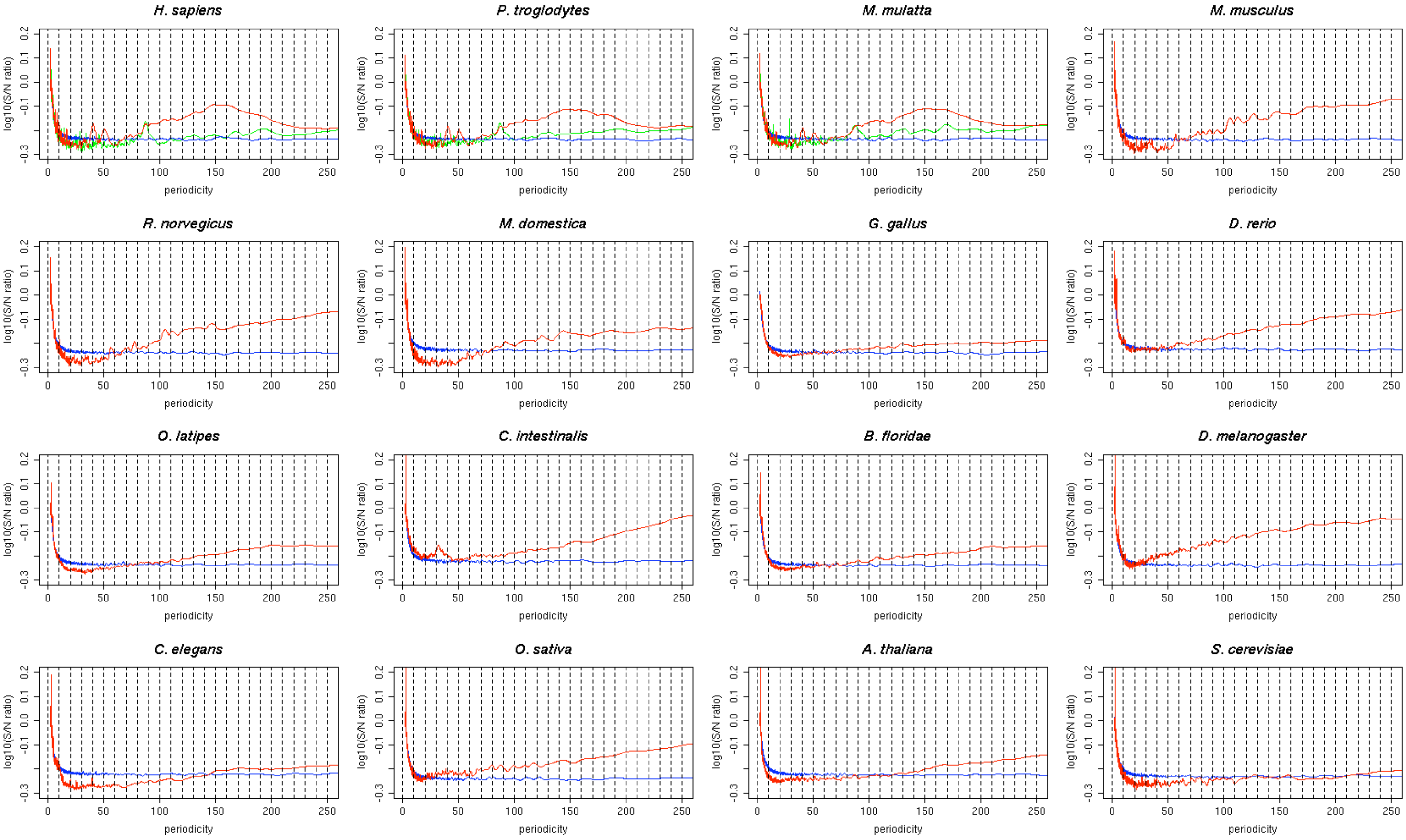
H. sapiens
P. troglodytes
M. mulatta
M. musculus
R. norvegicus
M. domestica
G. gallus
D. rerio
O. latipes
C. intestinalis
B. floridae
D. melanogaster
C. elegans
O. sativa
A. thaliana
S. cerevisiae
log10(S/N ratio)
periodicity
0.2
0.1
0.0
-0.1
-0.3
0
50
100
150
200
250

E. GC step

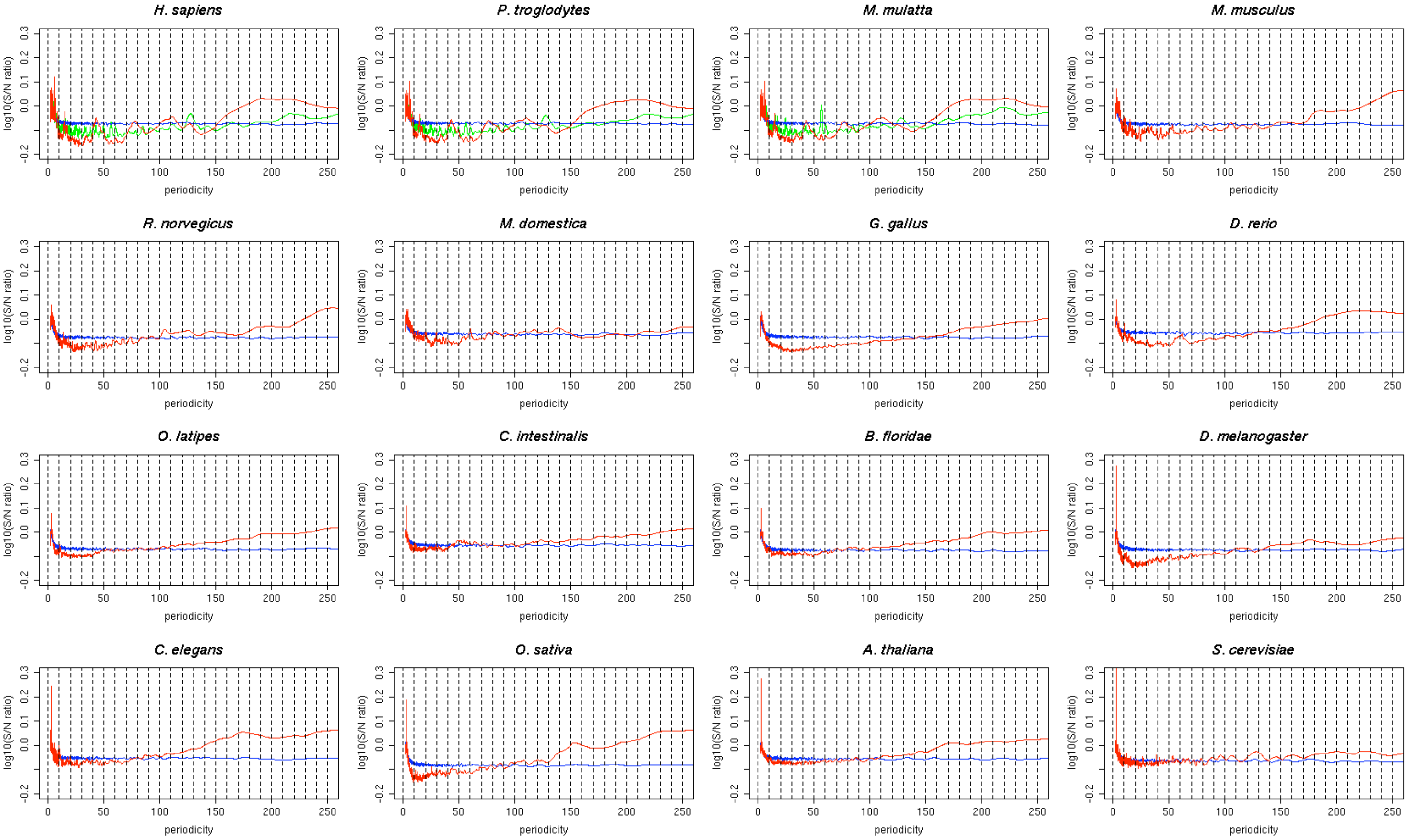

H. sapiens
P. troglodytes
M. mulatta
M. musculus
R. norvegicus
M. domestica
G. gallus
D. rerio
O. latipes
C. intestinalis
B. floridae
D. melanogaster
C. elegans
O. sativa
A. thaliana
S. cerevisiae
log10(S/N ratio)
periodicity
-0.2 0.0 0.1 0.2 0.3
0 50 100 150 200 250

F. GG/CC step

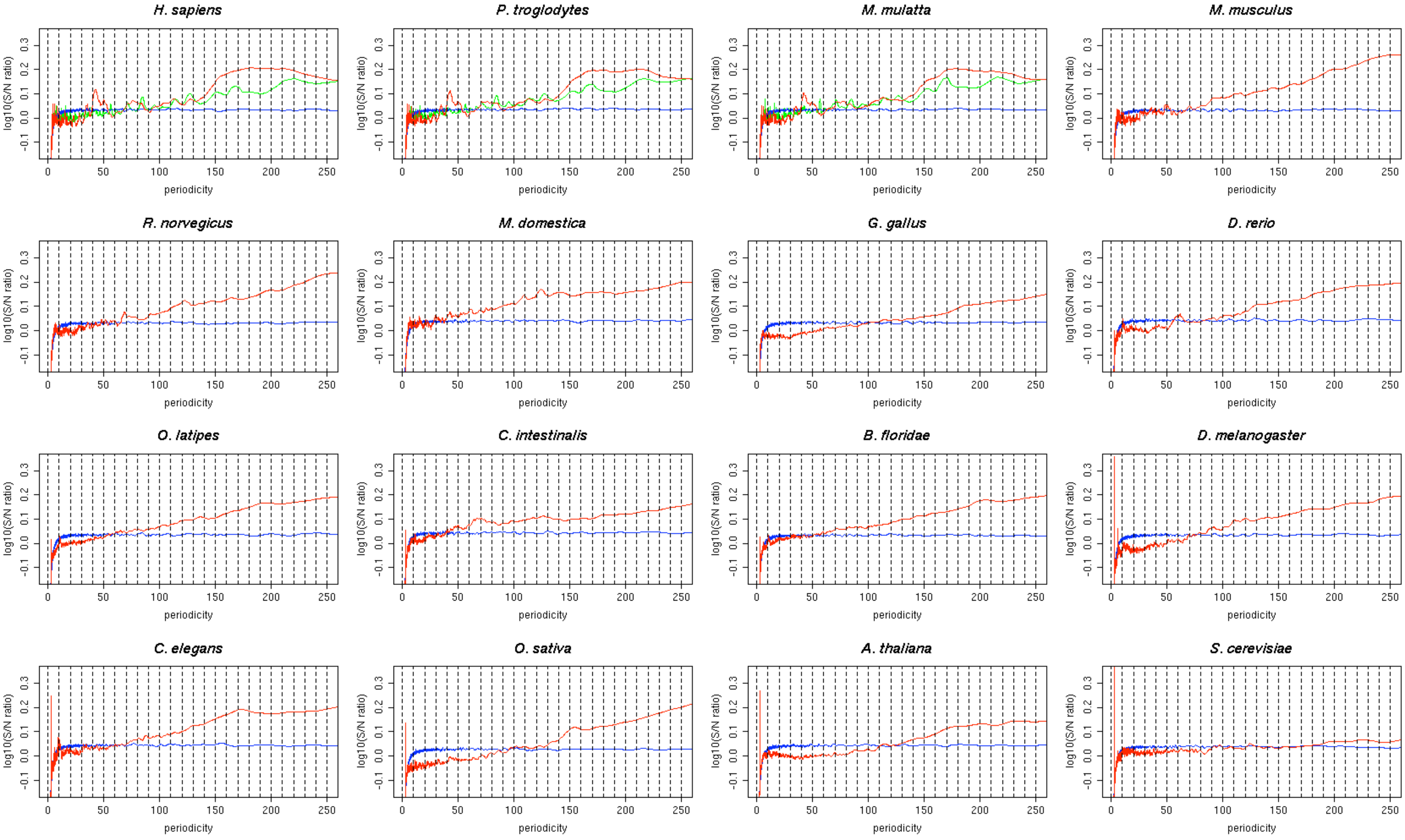

H. sapiens
P. troglodytes
M. mulatta
M. musculus
R. norvegicus
M. domestica
G. gallus
D. rerio
O. latipes
C. intestinalis
B. floridae
D. melanogaster
C. elegans
O. sativa
A. thaliana
S. cerevisiae
log10(S/N ratio)
-0.1
0.0
0.1
0.2
0.3
periodicity
0
50
100
150
200
250

G. GT/AC step

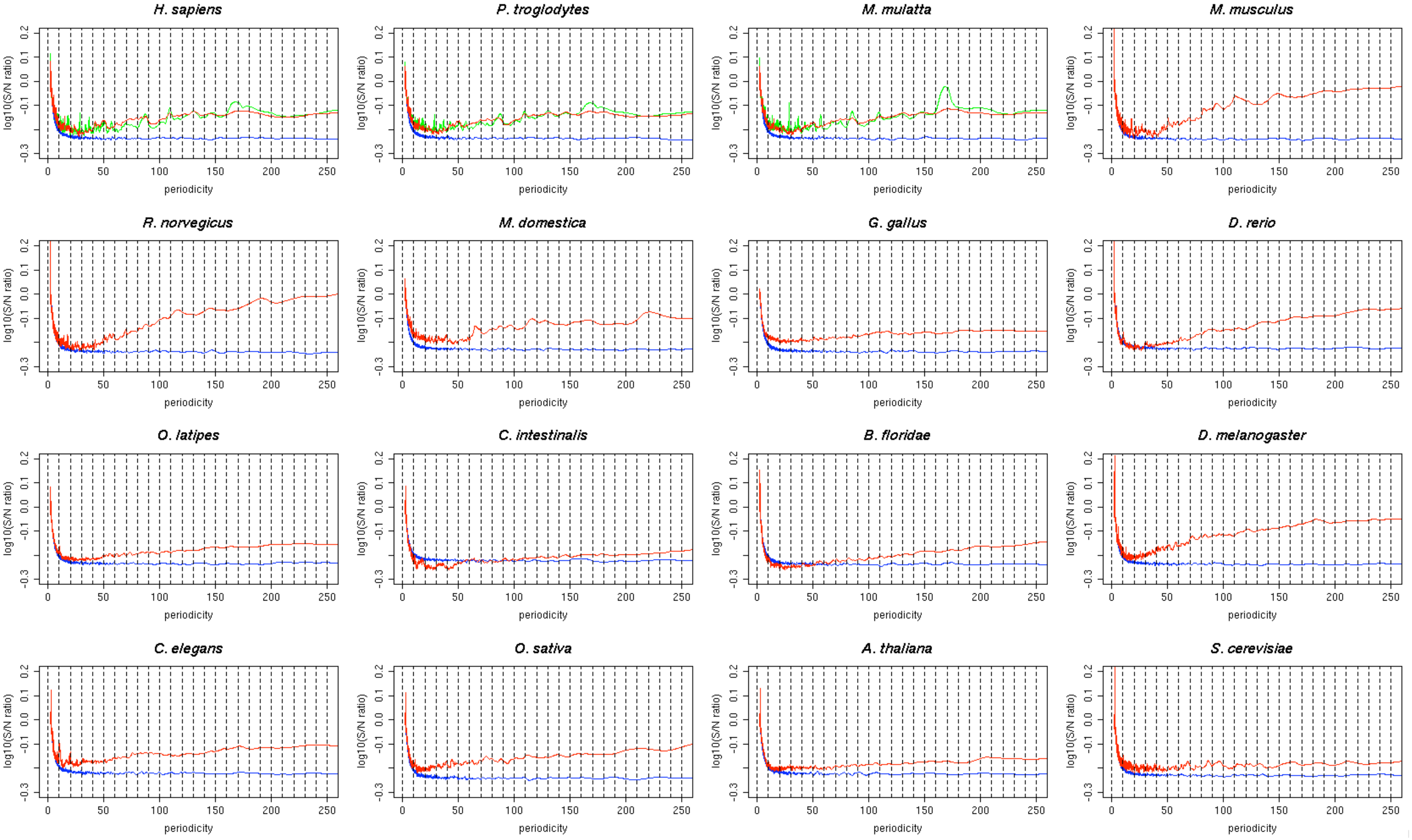
H. sapiens
P. troglodytes
M. mulatta
M. musculus
R. norvegicus
M. domestica
G. gallus
D. rerio
O. latipes
C. intestinalis
B. floridae
D. melanogaster
C. elegans
O. sativa
A. thaliana
S. cerevisiae
log10(S/N ratio)
periodicity

H. TA step

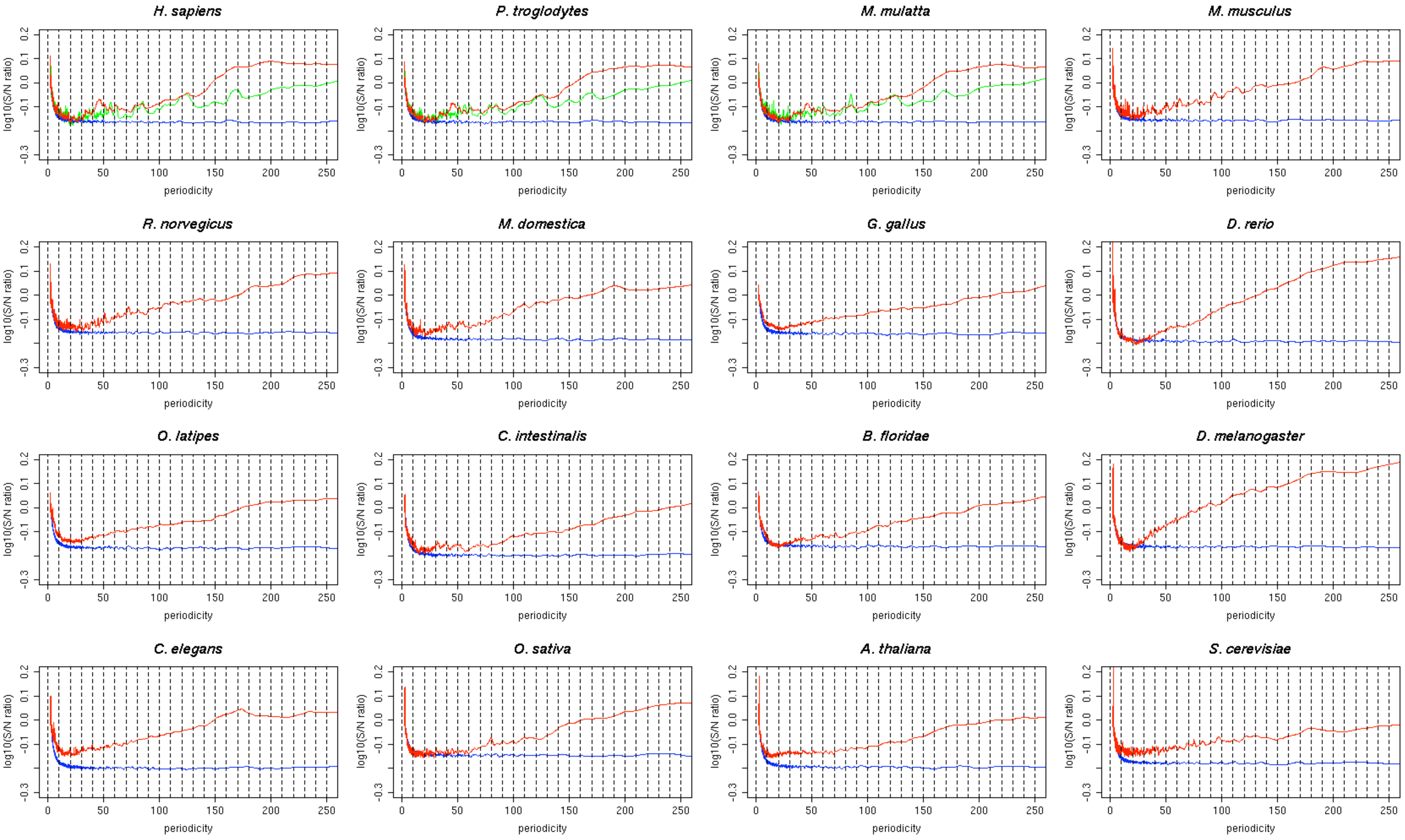
H. sapiens
P. troglodytes
M. mulatta
M. musculus
R. norvegicus
M. domestica
G. gallus
D. rerio
O. latipes
C. intestinalis
B. floridae
D. melanogaster
C. elegans
O. sativa
A. thaliana
S. cerevisiae
log10(S/N ratio)
periodicity
-0.3
-0.1
0.0
0.1
0.2
0
50
100
150
200
250

I. TG/CA step

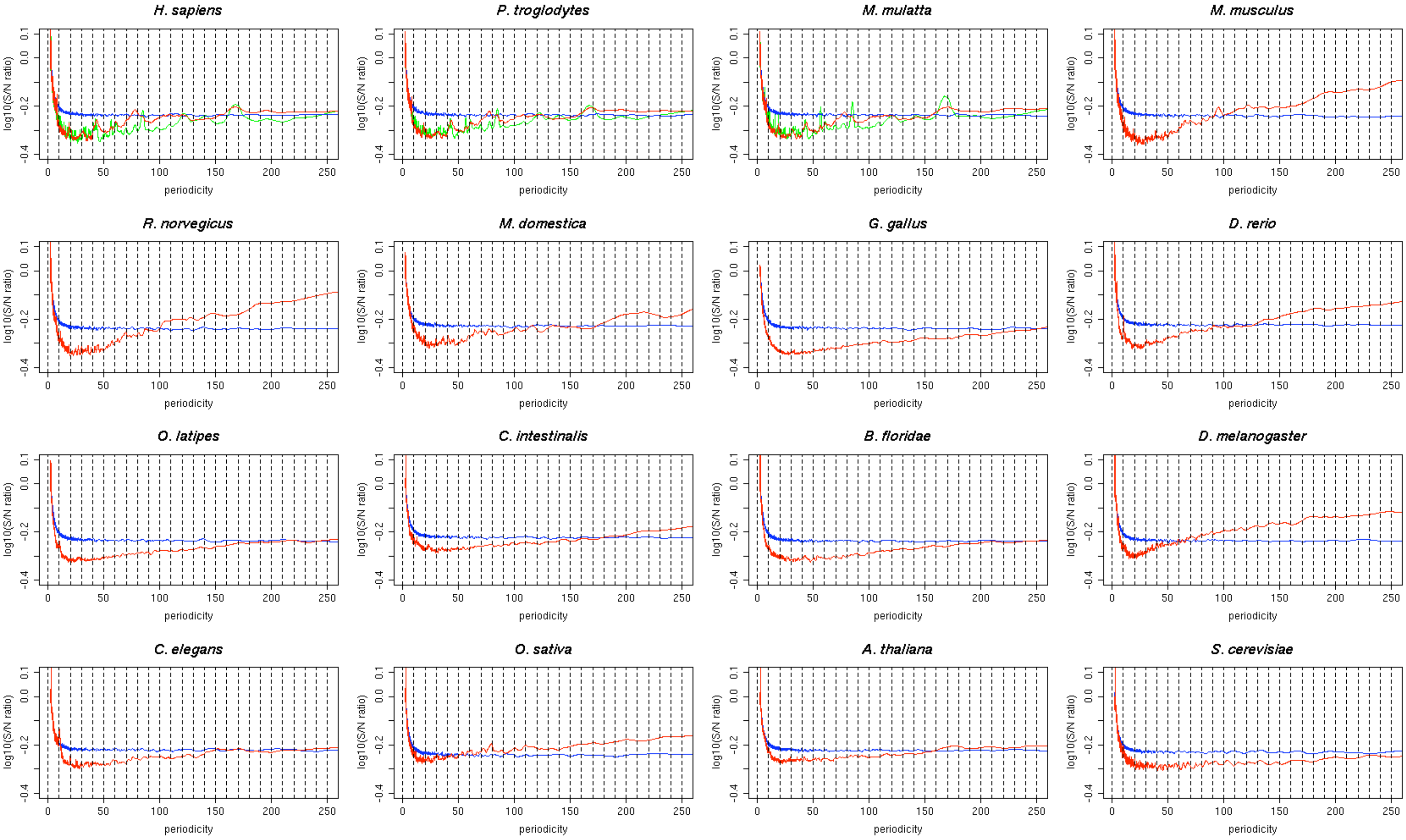
H. sapiens
P. troglodytes
M. mulatta
M. musculus
R. norvegicus
M. domestica
G. gallus
D. rerio
O. latipes
C. intestinalis
B. floridae
D. melanogaster
C. elegans
O. sativa
A. thaliana
S. cerevisiae
log10(S/N ratio)
periodicity
0.1
0.0
-0.2
-0.4
0
50
100
150
200
250

J. A/T step

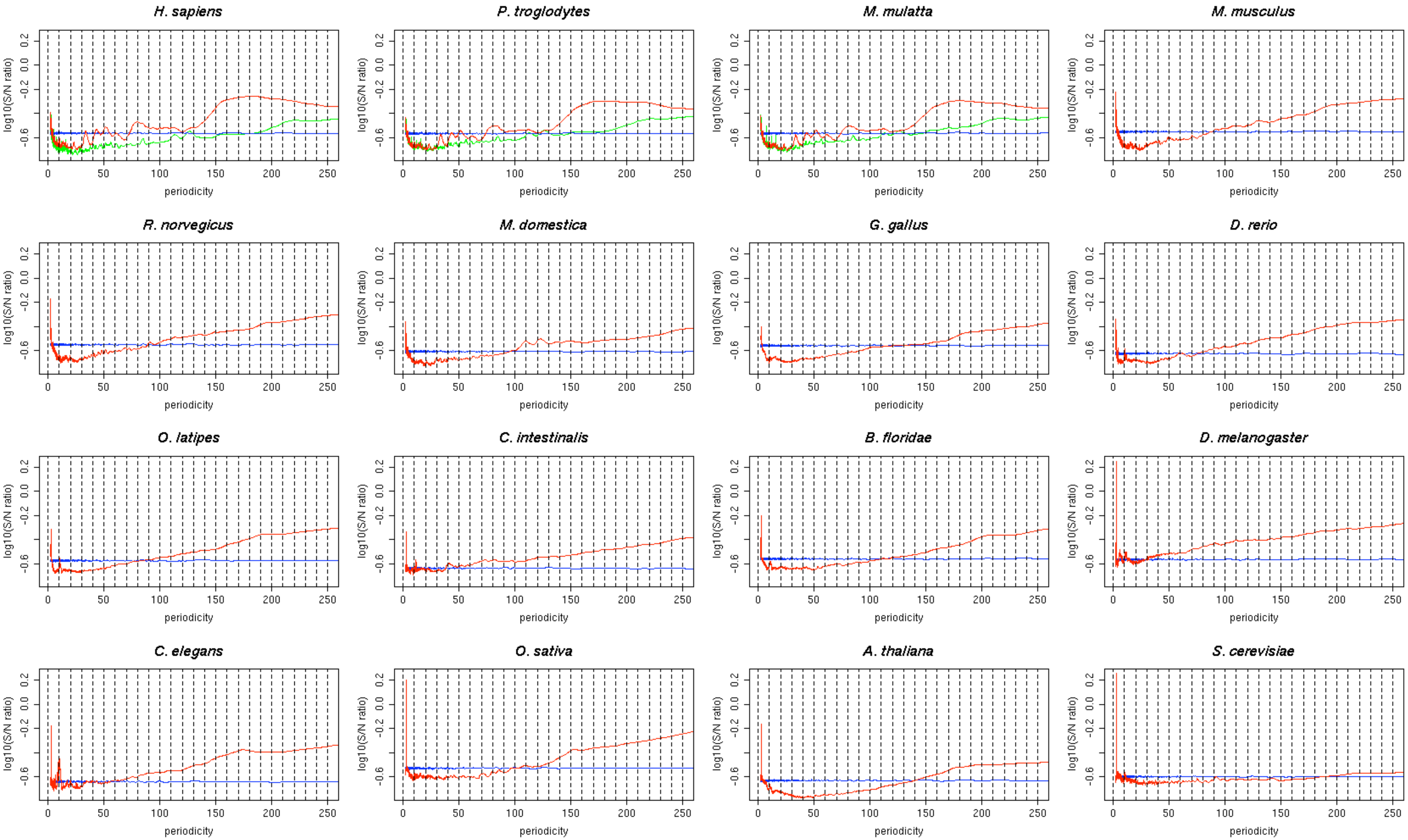
H. sapiens
P. troglodytes
M. mulatta
M. musculus
R. norvegicus
M. domestica
G. gallus
D. rerio
O. latipes
C. intestinalis
B. floridae
D. melanogaster
C. elegans
O. sativa
A. thaliana
S. cerevisiae
log10(S/N ratio)
periodicity
0.2
0.0
-0.2
-0.6
0
50
100
150
200
250

K. G/C step

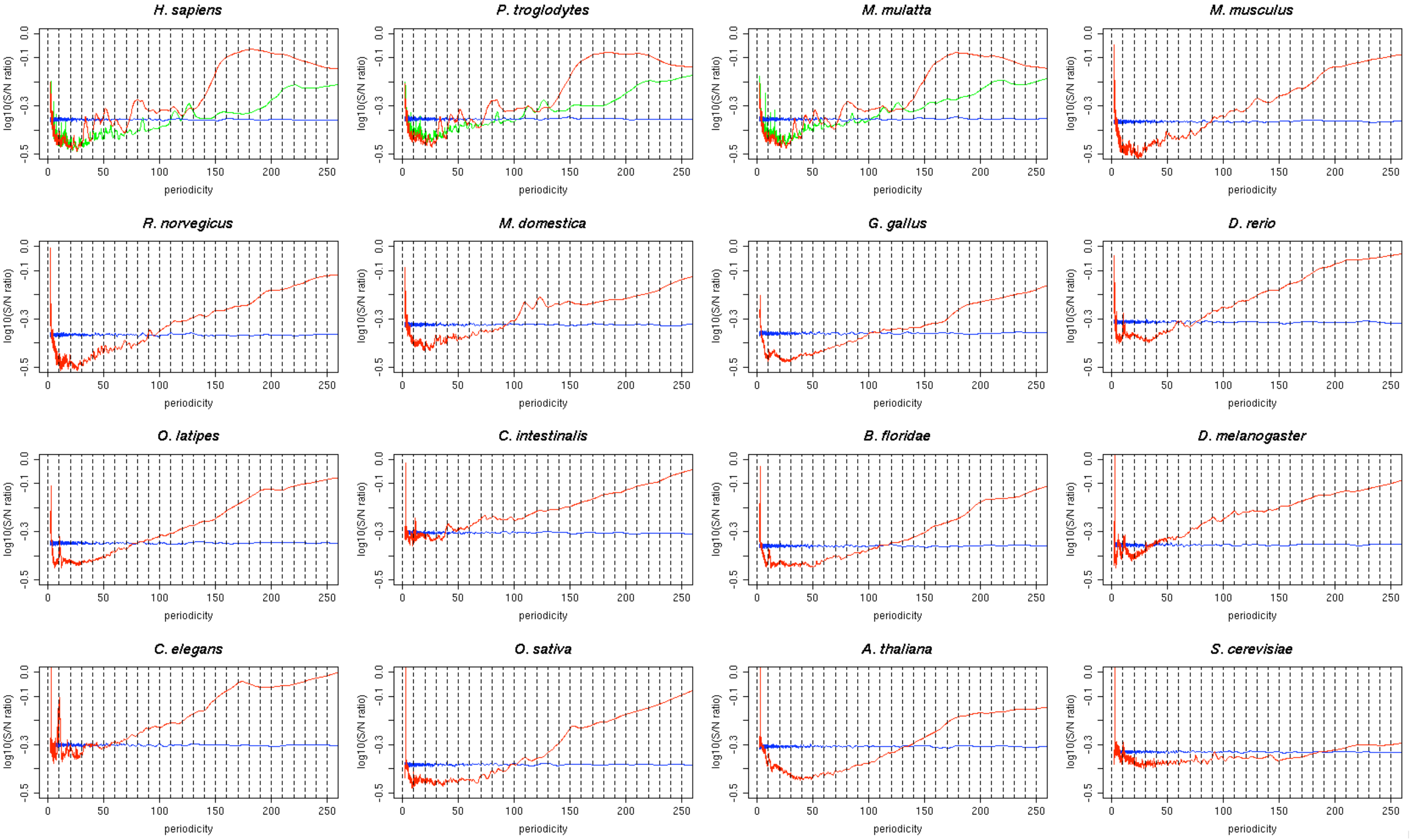
H. sapiens
P. troglodytes
M. mulatta
M. musculus
R. norvegicus
M. domestica
G. gallus
D. rerio
O. latipes
C. intestinalis
B. floridae
D. melanogaster
C. elegans
O. sativa
A. thaliana
S. cerevisiae
log10(S/N ratio)
0.0
-0.1
-0.3
-0.5
periodicity
0
50
100
150
200
250
