# Supplementary figures and images for: Effects of Alu elements on global nucleosome positioning in the human genome
Source: BMC Genomics. 2010 May 17;11:309. doi: 10.1186/1471-2164-11-309 (PMC2878307; doi:10.1186/1471-2164-11-309)

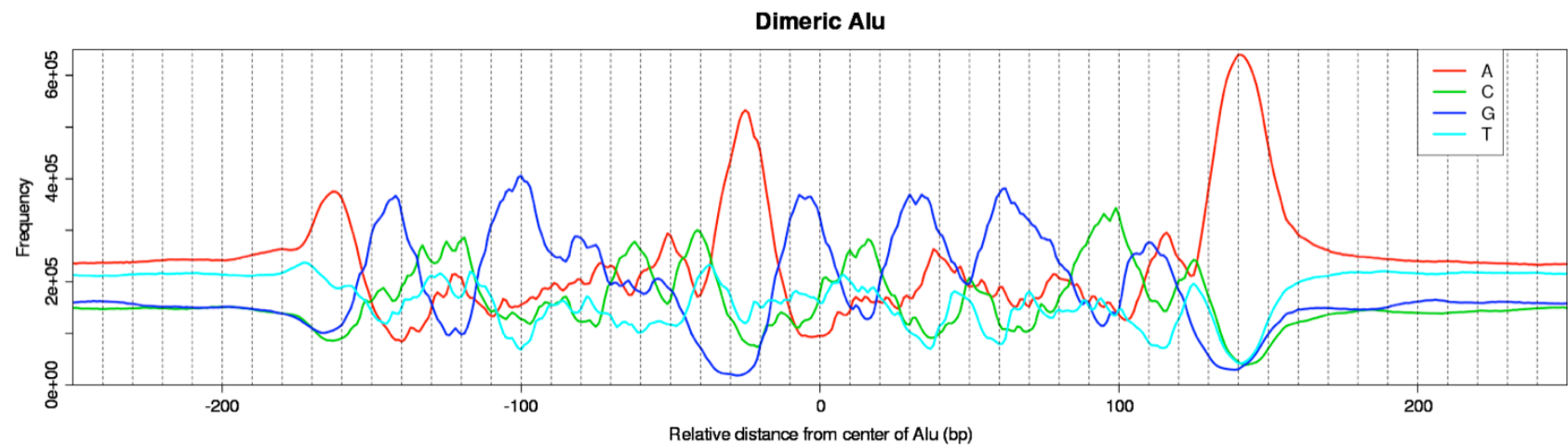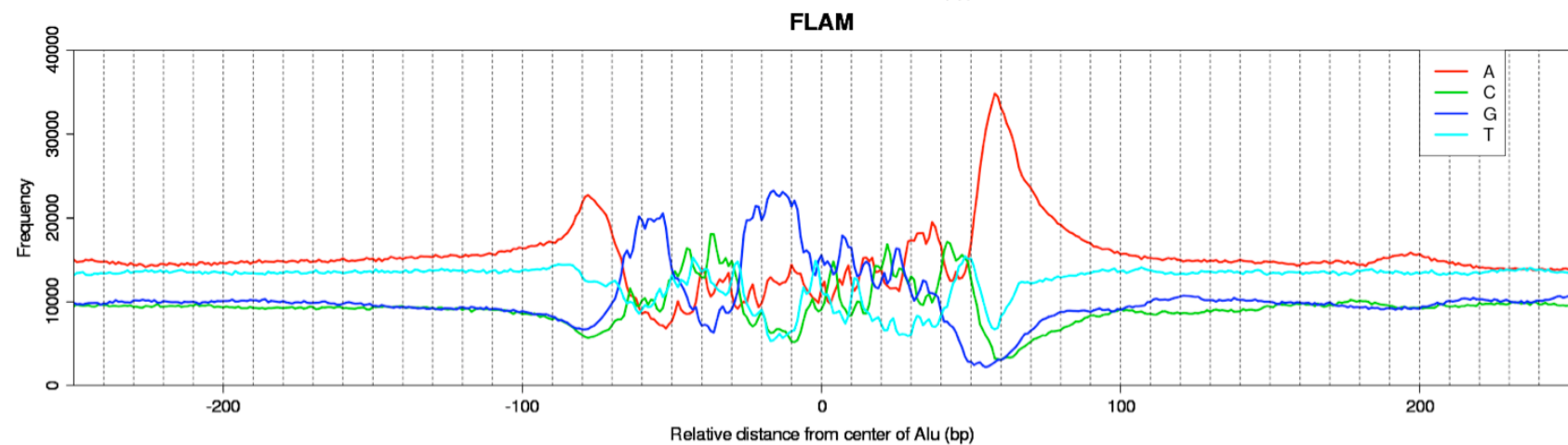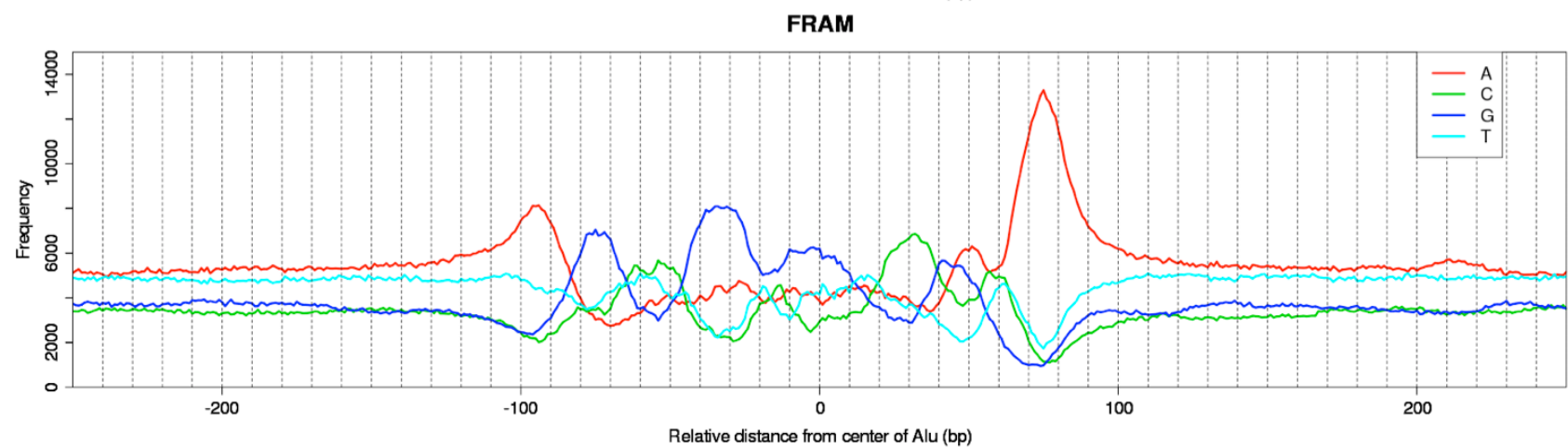

Supplement: Additional file 4 — Frequency of mononucleotides within and around Alu elements. Distribution of mononucleotides within and around Alu elements is shown. Red, green, blue and cyan lines represent the frequency of adenine, cytosine, guanine and thymine, respectively. [file 1471-2164-11-309-S4.PDF]

100%

Dimeric Alu

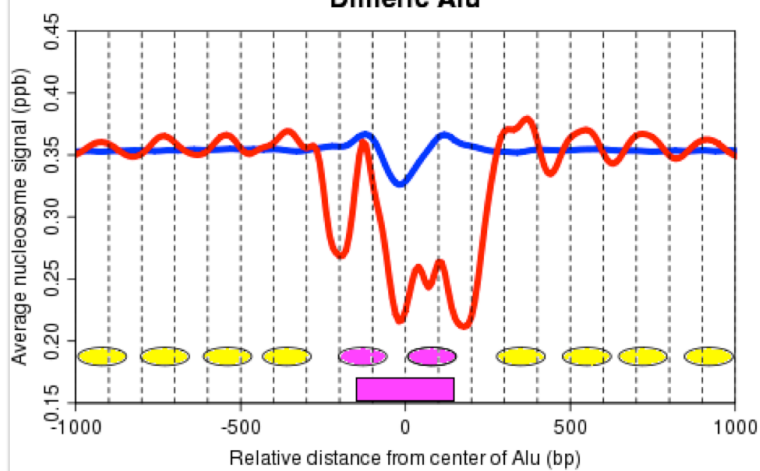

85%

Dimeric Alu

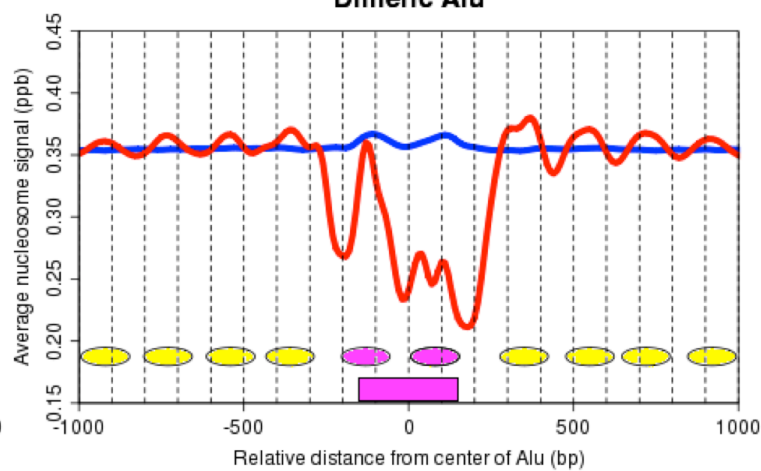

FLAM

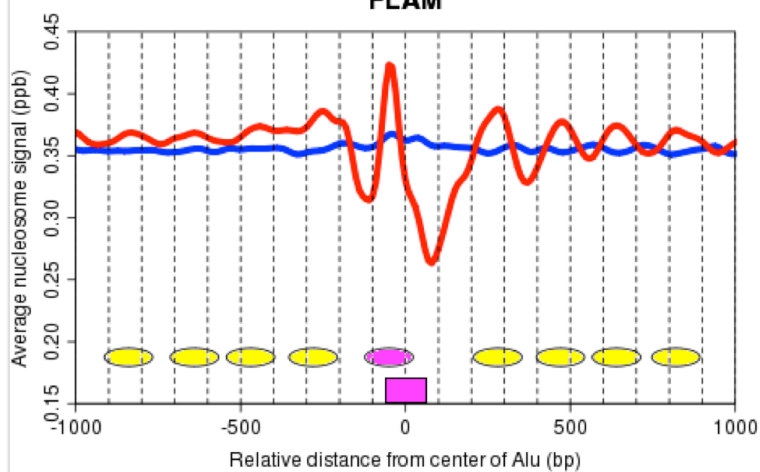

FLAM

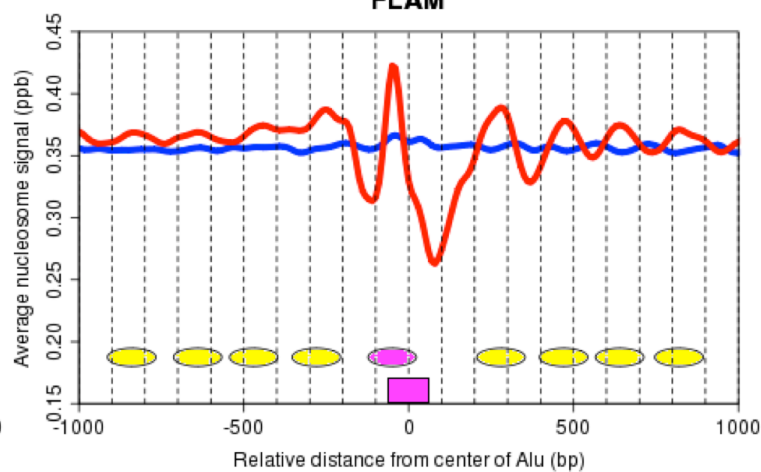

FRAM

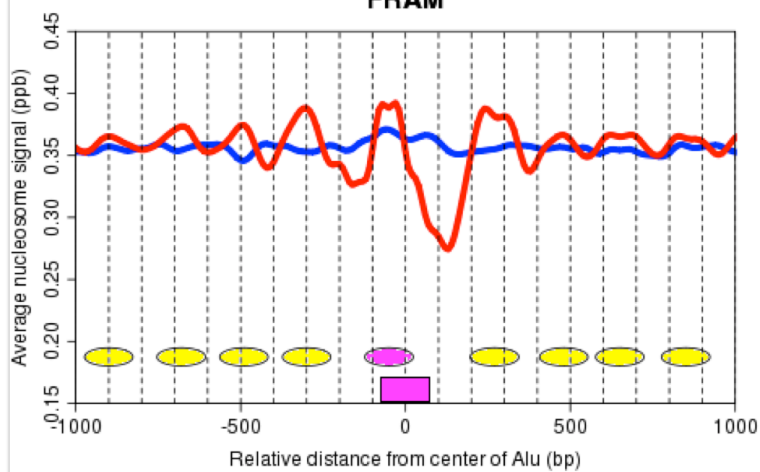

FRAM

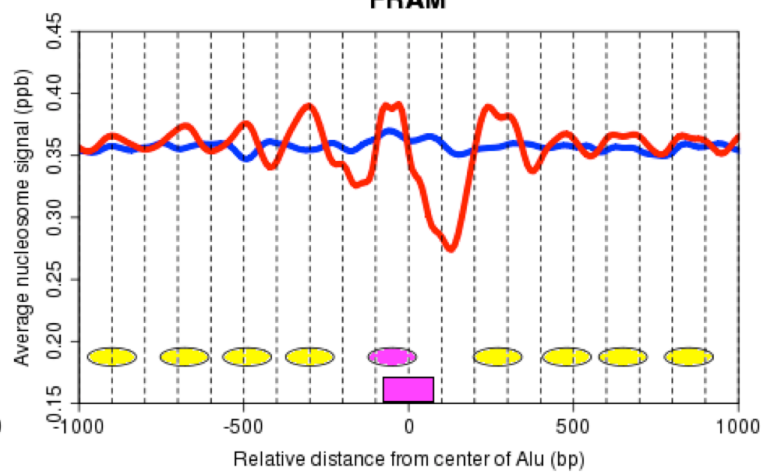

Supplement: Additional file 5 — Nucleosome distribution around Alu elements with multi-hit tags considered. Nucleosome distribution around dimeric Alu elements, FLAMs, and FRAMs are shown. In this data, multi-hit tags are included. [file 1471-2164-11-309-S5.PDF]

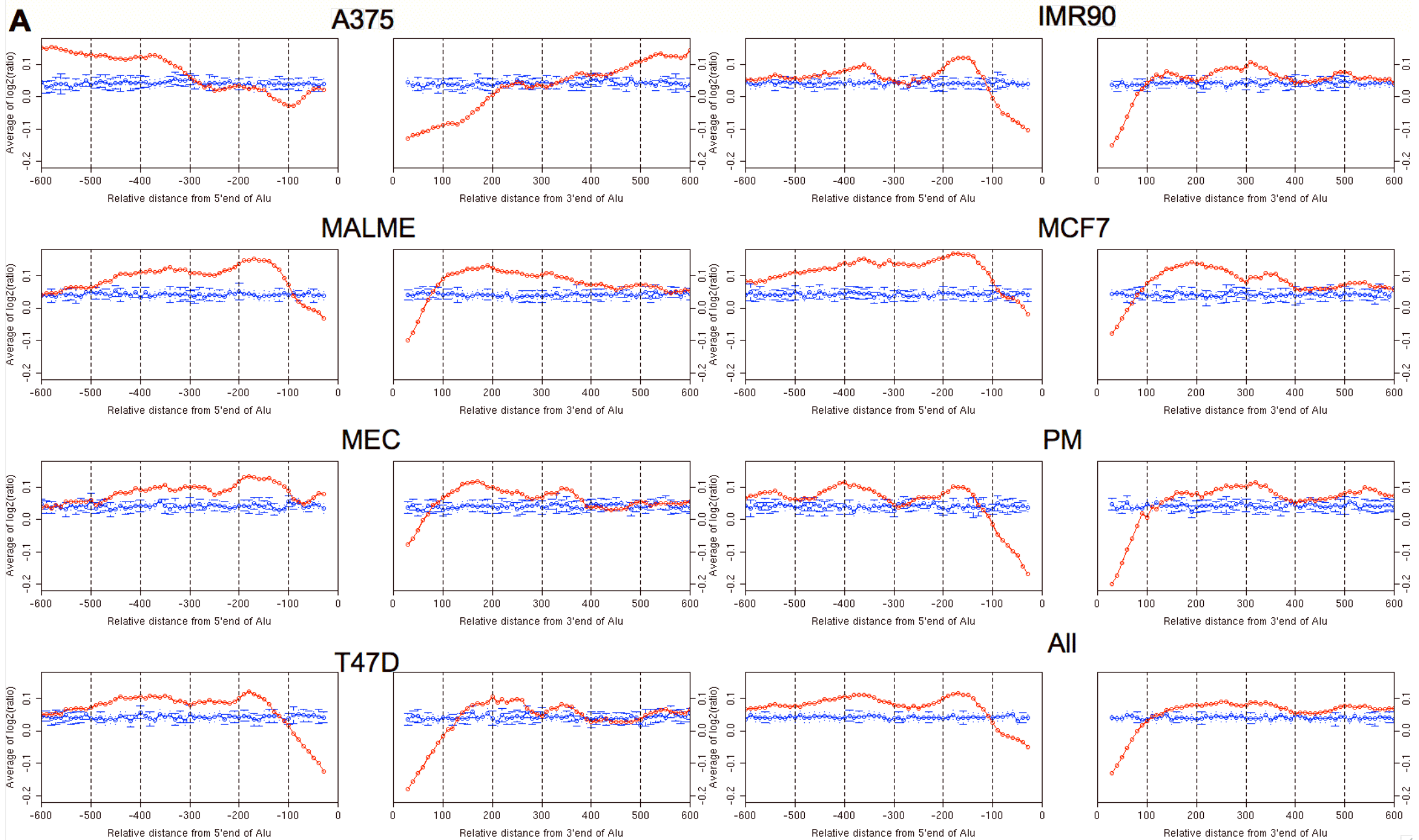

**B**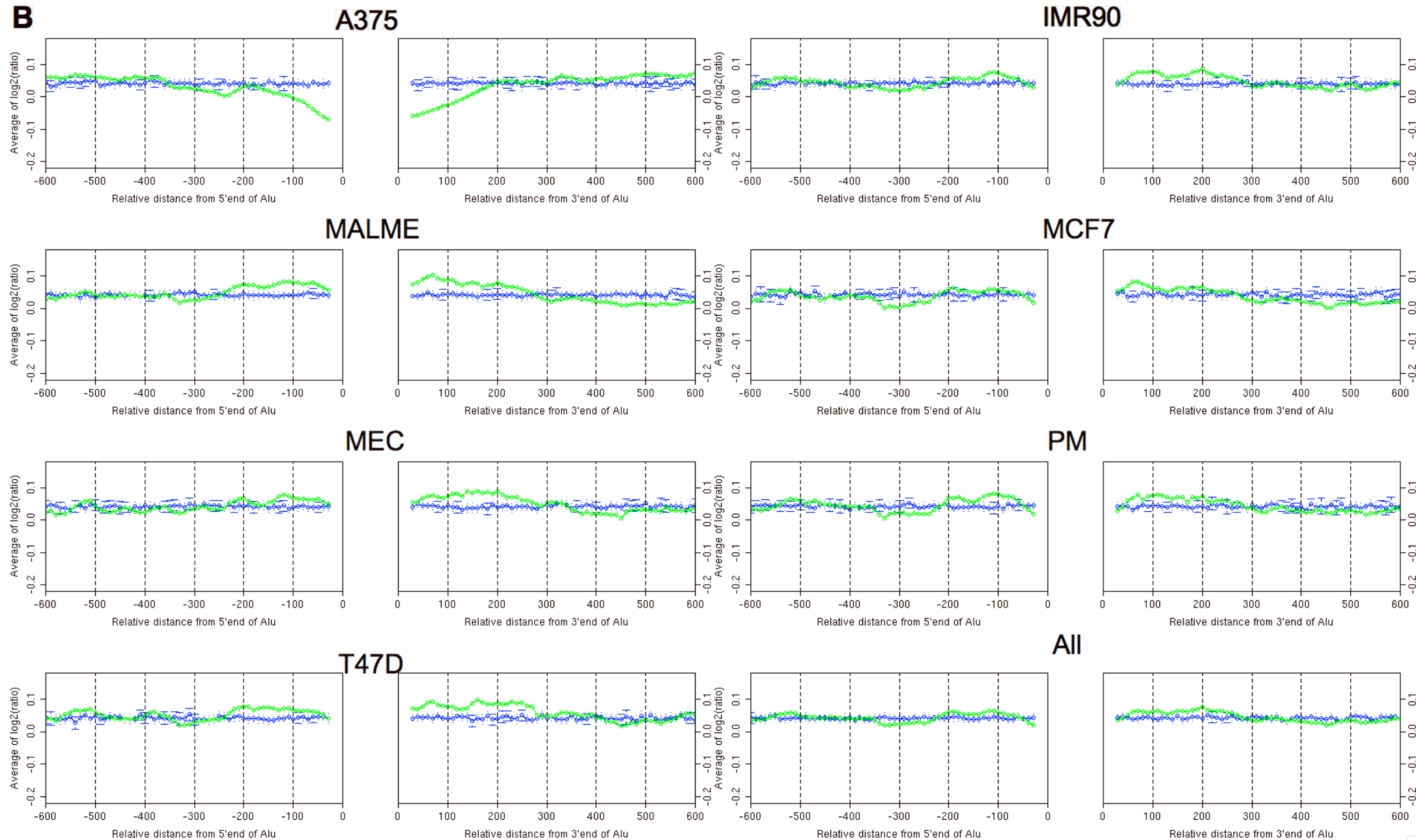

Supplement: Additional file 6 — Distributions of hybridization signals around (A) Alu elements and (B) other repetitive elements. In each cell type (A375, IMR90, MALME, MCF7, MEC, PM or T47D) or in all cells, average hybridization signals around repetitive elements are shown. [file 1471-2164-11-309-S6.PDF]

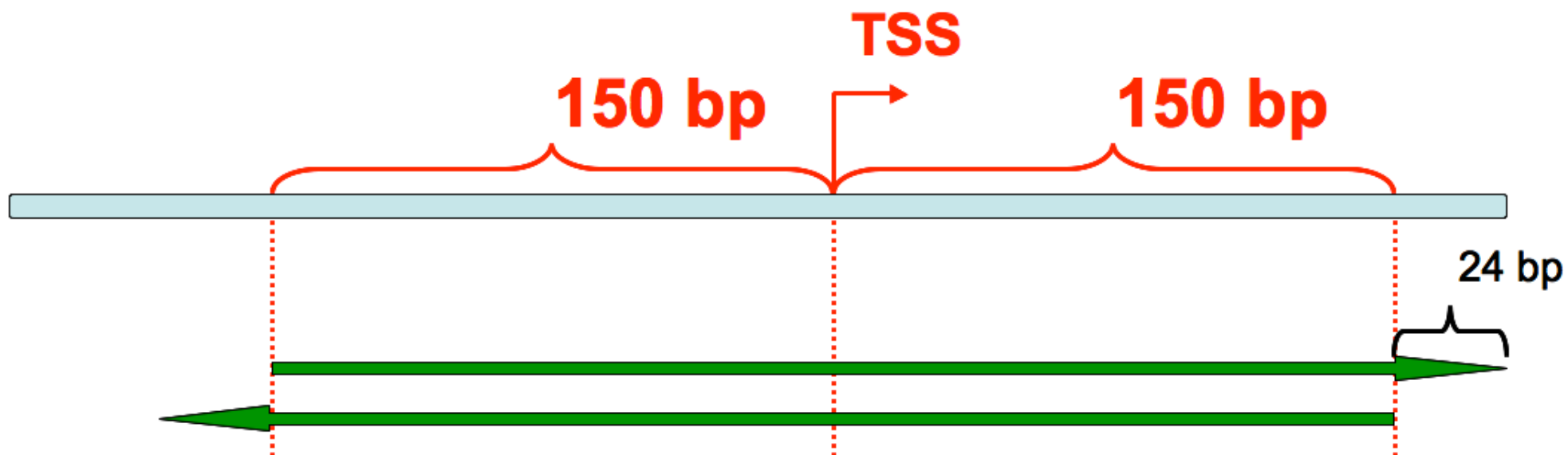

Supplement: Additional file 8 — Strategy of detection of sequence tags within ± 150 bp of TSSs. Green arrows represent DNA sequences used as reference sequences. The orientation of the arrow shows the strand. [file 1471-2164-11-309-S8.PDF]
